# Analysis of Microbiological Findings on the Surface of External Fixator Pins Comparing Steel Pins with Hydroxyapatite-Coated Pins

**DOI:** 10.1055/s-0045-1809337

**Published:** 2025-06-23

**Authors:** Cristhopher Stoffel, Honório Octávio Cuadro Peixoto, Felipe Kowaleski dos Santos, Pedro Afonso Keller Licks, Fernando Baldy dos Reis, Mauro José Costa Salles

**Affiliations:** 1 Instituto de Ortopedia e Traumatologia, Hospital São Vicente de Paulo, Passo Fundo, RS, Brazil; 2 Department of Orthopedics and Traumatology, Escola Paulista de Medicina, Universidade Federal de São Paulo, SP, Brazil; 3 Infectious Disease Discipline, Musculoskeletal Infection Group, Escola Paulista de Medicina, Universidade Federal de São Paulo, SP, Brazil

**Keywords:** bone nails, bone wires, external fixators, hydroxyapatite, infections, fios ortopédicos, fixadores externos, hidroxiapatita, infecções, pinos ortopédicos

## Abstract

**Objective:**

To compare the microbial retrieval rates and the organism types on the surface of stainless-steel pins (SSPs) and hydroxyapatite-coated pins (HCPs) from external fixators (EFs).

**Methods:**

The present prospective, non-randomized, multicenter, comparative interventional cohort study occurred from April 2018 to October 2021. The sample consisted of 44 patients with EFs, including 33 with SSPs and 11 with HCPs. We collected two pins from each patient, the one with the best and the one with the worst clinical appearance according to the Maz-Oxford-Nuffield (MON) classification, in an aseptic manner, and sent them for microbiological analysis.

**Results:**

The overall superficial infection (SI) rate was 52.3% (23 of 44 patients), affecting 45.5% (5 of 11) patients with HCPs and 54.5% (18 of 33) patients with SSPs ( *p* = 0.732). Of the 88 pins, 43.2% (38 of 88 pins) yielded microbial identification, with 42 pathogens isolated. *Staphylococcus aureus* was the most frequent organism, accounting for 59.5% (25 of 42 pathogens) of the positive samples. In the best-looking pins, the microbial retrieval rate was significantly lower in HCPs than SSPs, with 18.2% (2 pathogens in 11 pins) and 45.5% (15 pathogens in 33 pins), respectively ( *p* = 0.036). In the worst-looking pins, the microbial retrieval rate in HCPs and SSPs was 27.3% (3 pathogens in 11 pins) and 54.5% (18 pathogens in 33 pins), respectively ( *p* = 0.036).

**Conclusion:**

Microbial retrieval rates were lower in HCPs than in SSPs. However, these differences did not impact clinical infection rates, which were similar in both groups.

## Resumo

**Objetivo:**

Comparar as taxas de recuperação microbiana e os tipos de microrganismos identificados na superfície dos pinos de aço inoxidável (PAIs) e nos pinos revestidos com hidroxiapatita (PHAs) de fixadores externos (FEs).

**Métodos:**

Este estudo de coorte prospectiva de intervenção, não randomizado, multicêntrico, comparativo foi realizado entre abril de 2018 e outubro de 2021, com 44 pacientes tratados com FE, 33 dos quais receberam PAIs e 11 receberam PHAs. Foram coletados e enviados para análise microbiológica dois pinos de cada paciente, o de melhor e o de pior aspecto clínico conforme a classificação de Maz-Oxford-Nuffield (MON), de forma asséptica.

**Resultados:**

A taxa de infecção (TI) superficial global foi de 52,3% (23 de 44 pacientes), sendo 45,5% (5 de 11 pacientes) entre pacientes que receberam PHAs e 54,5% (18 de 33 pacientes) entre pacientes que receberam PAI, respectivamente ( *p* = 0,732). Dos 88 pinos, 43,2% (38 de 88 pinos) apresentaram identificação microbiana, sendo isolados 42 patógenos no total. O *Staphylococcus aureus* foi o mais frequente, representando 59,5% (25 dos 42 patógenos). Nas amostras de “melhor aspecto,” a taxa de recuperação microbiana foi significativamente menor nos PHAs do que nos PAIs, 18,2% (2 patógenos em 11 pinos) e 45,5% (15 patógenos em 33 pinos), respectivamente ( *p* = 0,036). Nas amostras de “pior aspecto,” a taxa de recuperação microbiana nos PHAs e nos PAIs foi 27,3% (3 patógenos em 11 pinos) e 54,5% (18 patógenos em 33 pinos), respectivamente ( *p* = 0,036).

**Conclusão:**

As taxas de recuperação microbiana foram menores nos PHA comparadas às dos PAI. Entretanto, estas diferenças não impactaram nas taxas de infecção clínica, que foram semelhantes nos dois grupos.

## Introduction

The external fixation method is widely used in the orthopedic field to treat fractures, pseudoarthrosis, and correct deformities. An external fixator (EF) is a metal device sustaining the rigidity or stability of the bone structure through transosseous wires or pins applied percutaneously. It usually consists of stainless steel, titanium, or metal alloys due to these materials' mechanical resistance and ability to withstand loads during bone healing. In addition to the base material, surface coating with other compounds, such as hydroxyapatite or silver, can improve the fixator's performance. Despite all potential uses, EFs are subject to complications, including surgical wound infection, pin loosening, and pin tract infection. 1 2 3

The most common complication is pin insertion site infection, especially in EFs used for a long time, with an incidence ranging from 11.3 to 100% of cases. 4 5 The current definition of tract infection consists of inflammatory signs around the pins, which require antibiotic administration, wire or pin removal, or surgical debridement. 6 Several hypotheses have been proposed for its pathophysiology. However, it is consensual that progressive inflammation in the presence of organisms alters the tissue microenvironment and reduces the immune system's ability to withstand bacterial proliferation. In addition, bacteria can adhere to the implant and form a biofilm by producing an extracellular matrix. 4 7

A biofilm is a three-dimensional, multicellular, and metabolically less active structure adhered to dead bone or the implant. 8 Biofilm formation hinders the action of antibiotics by restricting their entry through the extracellular matrix. Moreover, biofilm-associated bacteria present different physiological activity than that of free organisms, have an abnormal gene expression pattern, show slow and stationary growth, and develop in oxygen-deprived areas. 9 Due to these mechanisms, infection treatment may require high doses of antibiotics for a prolonged period and debridement or removal of the orthopedic implant. 8

In the literature, *Staphylococcus aureus* is the most commonly reported pathogen in pin tract infections, followed by *Staphylococcus epidermidis* , *Pseudomonas aeruginosa* , *Proteus mirabilis* , *Escherichia coli* , and *Corynebacterium* spp., among other organisms. 5 10 11 Therefore, in cases requiring empirical antibiotic treatment, anti-staphylococcal drugs are warranted until culture results are available. 5 However, the presence of biofilms may alter the antimicrobial action profile. In an in vitro study, monotherapy with high daptomycin doses had a bactericidal action against methicillin-resistant *S. aureus* (MRSA) strains, but only combined therapy with linezolid could sustain bactericidal activity against the biofilm of the same strain. 12 In another study in an animal model, systemic amikacin administration and implant impregnation with clarithromycin prevented the formation of *P. aeruginosa* biofilm. 13 Apparently, there is no correlation between the clinical picture and the infectious agent. 10

Strategies for infection prevention include coated pins or pins from different materials, 11 14 15 daily care around the pins with crust removal, and cleaning with chlorhexidine, iodine, or saline solutions. 4 7 16 17 Pin coating with hydroxyapatite is one of the most studied systems as it has osteoconductive properties and improves pin-bone fixation. Nevertheless, a recent prospective study showed no impact on superficial and deep infection rates associated with EF pins. 18 A meta-analysis comparing stainless-steel pins (SSPs), hydroxyapatite-coated pins (HCPs), silver, and titanium showed no statistically significant difference in infection rates. 14

Existing studies usually evaluate and compare infection rates in superficial and deep tissues in contact with EF pins according to the type of pin coating. However, the literature lacks an analysis of the most common microbiota on the surface of these devices. The current study aimed to assess infection rates in tissues in contact with EF pins, describe the organisms colonizing EF pins, and compare microbial retrieval rates in SSPs and HCPs.

## Materials and Methods

The present prospective, non-randomized, multicenter, comparative intervention cohort study including patients undergoing surgical treatment with any EF type from April 2018 to October 2021 occurred in 2 tertiary hospitals specialized in orthopedic diseases. These patients underwent an initial study to compare the infection rates in the tracts of pins with hydroxyapatite coating or not. 18 The current study compared microbial retrieval rates and identified the pathogens from EF pins with and without hydroxyapatite coating. The Research Ethics Committee approved this study under number CAAE 84939418.6.0000.5342, and all participants signed the informed consent form (ICF).

We included patients who agreed to participate by signing the ICF concerning data use and who underwent surgical treatment with any EF type to correct deformities or treat fractures, osteomyelitis, and/or pseudoarthrosis with the expectation of maintaining the EF for a minimum of 3 weeks. Patients underwent prospective follow-up every 4 weeks or as needed for treatment. We excluded subjects lost to follow-up in less than 1 year and those remaining with the EF for less than 3 weeks.

Using the Maz-Oxford-Nuffield (MON) classification 19 ( Table 1 ), previously validated for EF pin-associated infections, we collected two pins from each patient at the time of EF removal: the one with the best clinical appearance and the one with the worst clinical appearance in the tissues surrounding it. Thus, we formed two groups: one with the best-looking pins and one with the worst-looking pins from each patient. We used the microbial retrieval rate to assess the frequency of positive microbiological tests. To calculate the overall infection rate, patients who presented any pin with grade 2 or higher in the MON classification were considered infected.

**Table 1:** Maz-Oxford-Nuffield (MON) classification

Minor grades	Signs and symptoms	Treatment
1. Clinically, it is not seen as an infection, but a reaction	Slightly erythematous, with mild discharge	Improve pin care and observe local changes
2. Clinically seen as skin infection at the pin site	Skin erythema, serous or purulent discharge, soft-tissue pain and tenderness, and able to mobilize with analgesia.	Improve pin care, swab for culture and sensitivity, and start oral anti-Staphylococcus antibiotic regimen.
3	Severe erythema plus pain and purulent discharge with edema. Unable to mobilize with analgesia. Periosteal reaction on X-ray? Pin loosening?	Intensive care at pin site. Intravenous antibiotic administration for 5 to 7 days; limb elevation; x-ray to exclude periosteal reaction and pin loosening, which, if present, leads to grade 4
**Major grades**		
4	Severe soft-tissue involvement > 1 pin, periosteal reaction present, look for osteolysis	No response to local treatment or antibiotics. Pin removal; intravenous antibiotic administration for 5 to 7 days; plan to place a new pin at a new stage
5	Severe soft-tissue involvement > 1 pin plus osteomyelitis	Pin removal, additional surgery for bone infection control, specimen collection for culture and sensitivity, and prolonged intravenous antibiotic administration.
6	Sequestrum +/− fistula formation during treatment with the device	Potential for fixator removal; debridement of sequestration and collection of samples for culture and sensitivity. Limb salvage treatment and prolonged intravenous antibiotic administration.
Following device removal 6B	Pain/tenderness over the old pin tract +/− fistula formation. Sequestration in the old pin tract on X-ray	Pin tract debridement; collection of samples for culture and sensitivity; intravenous antibiotic administration for 3 weeks after surgery followed by oral administration for 2 weeks

**Fig. 1:**
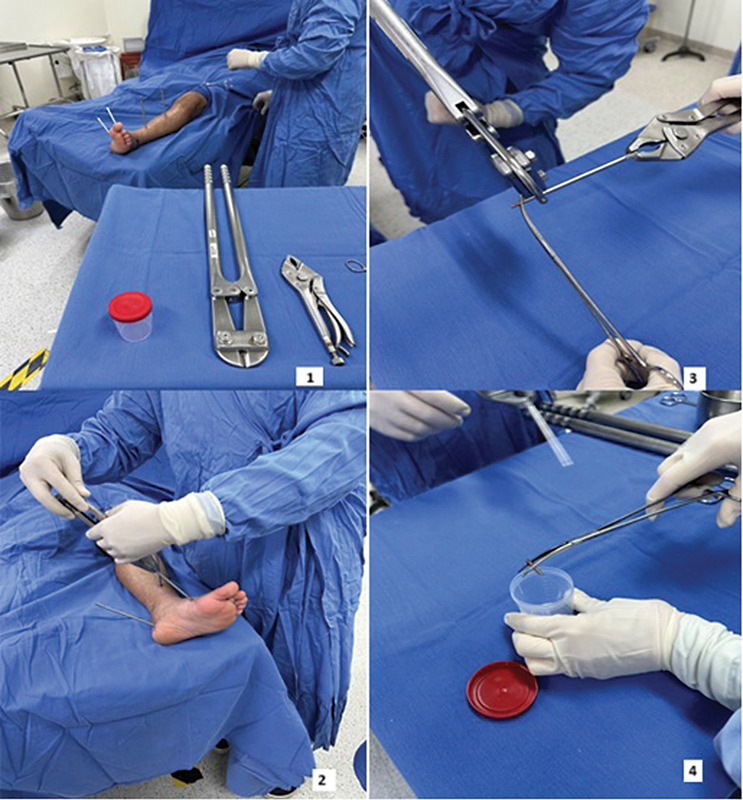
Method of aseptic collection of the pin intraosseous tip. ( **1** ) Asepsis and antisepsis with sterile field placement. ( **2** ) Pin removal. ( **3** ) Pin intraosseous tip section. ( **4** ) Sending the tip for culture.

We removed the pins from the EF aseptically, cut the intraosseous tips, and sent them for microbiological analysis in sterile and identified vials ( Fig. 1 ). We homogenized the samples in 3 mL of brain-heart infusion (BHI) broth and inoculated them in aerobic blood agar, chocolate agar, anaerobic blood agar, and thioglycolate broth. We incubated blood and chocolate agar plates at 35 to 37 C for 5 days (aerobic cultures) and 14 days (anaerobic cultures). We incubated thioglycolate broth for 14 days and, in case of bacterial growth, we placed the fluid on blood agar plates (aerobic and anaerobic cultures). Mass spectrometry identified the isolated bacterial colonies. The determination of the sensitivity profile from all strains occurred according to microbiological techniques standardized by the Clinical and Laboratory Standards Institute. 20

Statistical analysis was performed in IBM SPSS Statistics for Windows (IBM Corporation) version 27.0. We expressed categorical variables as absolute and relative frequencies. We assessed associations between variables using the Chi-squared test or Fisher's exact test when necessary. Statistical significance was set at *p* < 0.05.

## Results

Of all patients ( *n* = 44) included in this study, 33 were treated with EFs using SSPs and 11 with HCPs. The study population had 30 (68.2%) male and 14 (31.8%) female subjects. Considering the MON classification, the maximum grade in this sample was 3 ( Table 2 ). The overall infection rate was 52.3% (23 of 44 patients), with 45.5% (5 of 11) patients with HCPs and 54.5% (18 of 33) patients with SSPs ( *p* = 0.732).

**Table 2:** Characteristics from the study population ( *n* = 44)

	Hydroxyapatite ( *n* = 11)	Stainless steel ( *n* = 33)	*p*
**Male sex**	7 (63.6%)	23 (69.7%)	0.722
*Smoking*	—	5 (15.2%)	0.309
*Systemic hypertension*	1 (9.1%)	6 (18.2%)	0.659
*Diabetes mellitus*	—	2 (6.1%)	1.000
**Maximum Maz-Oxford-Nuffield grade**			
*1*	6 (54.5%)	15 (45.4%)	0.871
*2*	2 (18.2%)	7 (21.2%)	
*3*	3 (27.2%)	11 (33.3%)	
**Microbial retrieval rate**			
*Total number of patients*	3 (27.3%)	21 (63.4%)	0.036
*Worst-looking pin*	3 (27.3%)	18 (54.5%)	0.036
*Best-looking pin*	2 (18.2%)	15 (45.5%)	0.036

**Notes** : Values represent absolute and relative frequency; *P* , probability value (Chi-squared test).

The total number of pin cultures was 88 (2 pins from each patient: the best-looking and the worst-looking pins according to the MON classification). Bacterial retrieval occurred in 38 (43.2%) of the 88 pins evaluated, with 42 pathogens isolated. *S. aureus* was the most frequent among these 42 pathogens, appearing 25 (59.5%) times.

Of the 11 patients with HCPs, microbial retrieval occurred in 3 (27.3%). Of these 3 patients, 2 (66.6%) had Gram-positive bacteria, 1 (33.3%) had Gram-negative bacteria, and 1 (33.3%) had multidrug-resistant bacteria ( Tables 3 , 4 ). Of the 33 subjects with SSPs, microbial retrieval occurred in 21 (63.6%). Of these 21 patients, 16 (76.2%) had Gram-positive bacteria, 6 (28.6%) had Gram-negative bacteria, and 9 (42.3%) had multidrug-resistant bacteria ( Tables 3 , 4 ). The microbial retrieval rate was significantly higher in SSPs than in HCPs (63.4% [21 of 33 patients] versus 27.3% [3 of 11 patients] respectively, *p* = 0.036).

**Table 3:** Proportion of gram-positive, gram-negative, and multidrug-resistant organisms among colonized patients

	Hydroxyapatite		*p*
	Yes	No	
**Worst-looking pin**	n = 3	n = 21	
Gram-positive	2 (66.7%)	16 (88.9%)	0.489
Gram-negative	1 (33.3%)	5 (27.8%)	1.000
Multidrug-resistant	1 (33.3%)	9 (44.4%)	1.000
**Best-looking pin**	n = 3	n = 14	
Gram-positive	3 (100%)	12 (80.0%)	1.000
Gram-negative	0	3 (20.0%)	1.000
Multidrug-resistant	0	7 (46.7%)	0.485

**Note** : Values represent absolute and relative frequency.

**Table 4:** Proportion of gram-positive, gram-negative, and multidrug-resistant organisms among all patients

	Hydroxyapatite		*p*
	Yes ( *n* = 11)	No ( *n* = 33)	
**Worst-looking pin**			
Gram-positive	2 (18.2%)	15 (45.5%)	0.158
Gram-negative	1 (9.1%)	5 (15.2%)	1.000
Multidrug-resistant	1 (9.1%)	8 (24.2%)	0.411
**Best-looking pin**			
Gram-positive	2 (18.2%)	12 (36.4%)	0.456
Gram-negative	0	3 (9.1%)	0.561
Multidrug-resistant	0	7 (21.2%)	0.165

**Note** : Values represent absolute and relative frequency.

Analyzing the microbial retrieval rate from the 44 pins with the best clinical appearance, specifically 33 SSPs and 11 HCPs, microbial retrieval occurred in 15 SSPs (45.5%) and 2 HCPs (18.2%). In total, microbial retrieval occurred in 17 (38.6%) of the 44 best-looking pins ( Table 2 ). In the 44 worst-looking pins, that is, 33 SSPs and 11 HCPs, microbial retrieval occurred in 18 SSPs (54.5%) and 3 HCPs (27.3%). In total, microbial retrieval occurred in 21 (47.4%) of the 44 worst-looking pins ( Table 2 ). The microbial retrieval rate in uncoated pins was significantly higher than in hydroxyapatite-coated pins, and this was evident both in the sample of “best-looking” pins, respectively 45.5% (15 of 33 SSP) versus 18.2% (2 of 11 HCP), *p* = 0.036, and in the “worst-looking” pins, respectively 54.5% (18 of 33 SSP) versus 27.3% (3 of 11 HCP), with *p* = 0.036 ( Table 2 ). Of the 21 patients with microbial retrieval in the worst-looking pins, 14 (66.7%) also had a positive culture in the best-looking pins. Of the 17 patients with a positive culture in the best-looking pins, 14 (82.4%) also had a positive culture in the worst-looking pins.

Follow-up cultures revealed that *S. aureus* had the highest absolute frequency among pathogens, being found in 14 of the worst-looking pins and 11 of the best-looking pins, followed by *Serratia marcescens* , found in two of the best-looking pins and one of the worst-looking pins, and *Enterococcus faecalis* , found in two of the worst-looking pins and one of the best-looking pins. Next, came *Acinetobacter baumannii* , coagulase-negative *Staphylococcus* , and other bacteria described in Table 5 . Interestingly, two patients with no bacterial growth in the worst-looking pin had *S. epidermidis* and *Staphylococcus hominis* in the best-looking pin.

**Table 5:** Bacteria identified in culture during follow-up and respective absolute frequencies

	Worst-looking pin	Best-looking pin
*Acinetobacter baumannii*	1	1
*Bacillus sp.*	1	—
*Enterobacter cloacae*	1	—
*Enterococcus faecalis*	2	1
*Pseudomonas aeruginosa*	1	—
*Staphylococcus aureus*	14	11
*Staphylococcus epidermidis*	—	1
*Staphylococcus hominis*	—	1
*Serratia marcescens*	1	2
*Sphingomonas paucimobilis*	1	—
Coagulase-negative *Staphylococcus*	1	1
*Stenotrophomonas maltophilia*	1	—

**Note** : Values express absolute frequency.

The cultures provided clinically relevant information in addition to the MON classification. As described in Table 6 , approximately 1 in 5 patients were MON 1, that is, without clinical infection, had positive cultures. Furthermore, approximately half of the patients classified as MON 2 and 3, that is, with clinical infection, had negative cultures.

**Table 6:** Prevalence of organism retrieval per the Maz-Oxford-Nuffield (MON) classification ( *n* = 44 patients/88 pins)

	MON grade			*p*
	1 *(n = 21 / 42)*	2 *(n = 9 / 18)*	3 *(n = 14 / 28)*	
Worst-looking pin	5 (23.8%)	7 (77.8%)	9 (64.3%)	0.014
Best-looking pin	4 (19.0%)	3 (33.2%)	10 (71.4%)	0.024

**Notes** : Values represent absolute and relative frequency. *P* , probability value (Chi-squared test).

## Discussion

In the present study, the microbial retrieval rates in HCPs were significantly lower than those in SSPs. However, we found no statistical difference in the clinical infection rates, as reported by Stoffel et al. 14 18 Pizà et al. 11 also found no significant difference in the infection rates when comparing the tracts of HCPs and SSPs but demonstrated that hydroxyapatite-coated pins present higher pin-bone adhesion and better osseointegration, leading to lower pin loosening rates. Pieske et al. 21 concluded that better pin-bone adhesion in HCPs is clinically irrelevant because it does not reduce pin loosening or infection rates.

Regarding the microbial retrieval rate, our results differ from those usually found in the literature. Although in vivo studies are scarce, several of them concluded that hydroxyapatite is more prone to microbial adhesion and biofilm formation due to its rough and porous surface, which presents more binding sites for organisms. McEvoy et al. 22 found a slightly greater propensity for *P.* *mirabilis* and *S. epidermidis* to form biofilms on Kirschner wires coated with hydroxyapatite than stainless steel. Oga et al. 23 observed a similar result when evaluating discs from different materials (stainless steel, titanium alloy, and hydroxyapatite) using electron microscopy; these authors demonstrated higher adhesion of *S. epidermidis* on discs coated with hydroxyapatite compared with stainless steel. Ravn et al. 24 performed a microcalorimetric analysis of biofilms collected from materials with smooth surfaces (cobalt-chrome, titanium), porous surfaces (hydroxyapatite), and polyethylene; the group with porous surfaces showed the highest biofilm growth compared with the others. However, Arciola et al. 25 found significantly lower bacterial adhesion of *S. epidermidis* to HCPs in an in vitro study exposing the pins to bacterial solutions and incubating them in a culture medium. The authors identified that, in saline solution, hydroxyapatite releases calcium and phosphorus ions progressively over time, and this release coincides with a decrease in bacterial adhesion.

A rare in-vivo study 11 investigated the infection rate between HCPs and SSPs and found no statistical difference in the infection rate or the microbial retrieval rate, except for *P. aeruginosa* , which was more frequently isolated in HCPs, with no explanation for this finding. In our study, only one pin, also an HCP, had *P. aeruginosa* .

The microbiological profile identified in the present study corroborates the findings from other studies involving EFs. The most frequently identified bacteria in cultures were *S. aureus* , representing approximately 60% of the microbial retrieval, followed by *Serratia mercescens* , *E. faecalis* , and *Acinetobacter baumannii* . Pizà et al. 11 identified *S. aureus* as the etiological agent of pin tract infection in more than 50% of cases, followed by Gram-negative germs, such as *P. aeruginosa* , *P. mirabilis* , and *E. coli* . In a Swedish study 26 with 106 patients, at the end of prolonged treatment with EF, cultures from all pin tips revealed a positivity rate of 39%, with *S. aureus* accounting for 63% of these results.

Among the limitations of this study, we highlight the relatively small sample size of pins, the potential interference of antibiotic use in patients with EFs for a prolonged period, the lack of differentiation between patients treating infection, fracture, or deformity, and the lack of a median time analysis. However, it is known that HCPs are not often used in orthopedic damage control.

## Conclusion

The lower microbial retrieval rates in HCPs than in SSPs in both groups could suggest higher resistance to microbial adhesion on HCPs. However, confirmation of this hypothesis would require sonication of the extracted implants, which was not feasible. Nevertheless, these differences did not impact the clinical infection rates, which were similar in patients who used EFs with coated or uncoated pins. *S. aureus* accounted for most of the positive cultures.

## References

[1] Sisk T D General principles and techniques of external skeletal fixation Clin Orthop Relat Res 1983 180 96 100 6627801

[2] Bliven E K Greinwald M Hackl S Augat P External fixation of the lower extremities: Biomechanical perspective and recent innovations Injury 2019 50 01 S10 S17 10.1016/j.injury.2019.03.041 31018903

[3] Huiskes R Chao E Y Crippen T E Parametric analyses of pin-bone stresses in external fracture fixation devices J Orthop Res 1985 3 03 341 349 10.1002/jor.1100030311 4032105

[4] Jennison T McNally M Pandit H Prevention of infection in external fixator pin sites Acta Biomater 2014 10 02 595 603 10.1016/j.actbio.2013.09.019 24076071

[5] Checketts R G MacEachern A G Otterburn M Pin track infection and the principles of pin site care London Springer-Verlag 2000 97 103 10.1007/978-1-4471-0691-3_11

[6] Ceroni D Grumetz C Desvachez O Pusateri S Dunand P Samara E From prevention of pin-tract infection to treatment of osteomyelitis during paediatric external fixation J Child Orthop 2016 10 06 605 612 10.1007/s11832-016-0787-8 27848193 PMC5145837

[7] Parameswaran A D Roberts C S Seligson D Voor M Pin tract infection with contemporary external fixation: how much of a problem? J Orthop Trauma 2003 17 07 503 507 10.1097/00005131-200308000-00005 12902788

[8] Zimmerli W Sendi P Orthopaedic biofilm infections APMIS 2017 125 04 353 364 10.1111/apm.12687 28407423

[9] Ciofu O Rojo-Molinero E Macià M D Oliver A Antibiotic treatment of biofilm infections APMIS 2017 125 04 304 319 10.1111/apm.12673 28407419

[10] W-Dahl A Toksvig-Larsen S Lindstrand A No difference between daily and weekly pin site care Acta Orthop Scand 2003 74 06 704 708 10.1080/00016470310018234 14763702

[11] Pizà G Caja V L González-Viejo M A Navarro A Hydroxyapatite-coated external-fixation pins. The effect on pin loosening and pin-track infection in leg lengthening for short stature J Bone Joint Surg Br 2004 86 06 892 897 10.1302/0301-620x.86b6.13875 15330032

[12] Parra-Ruiz J Bravo-Molina A Peña-Monje A Hernández-Quero J Activity of linezolid and high-dose daptomycin, alone or in combination, in an in vitro model of Staphylococcus aureus biofilm J Antimicrob Chemother 2012 67 11 2682 2685 10.1093/jac/dks272 22796888

[13] Cirioni O Ghiselli R Silvestri C Minardi D Gabrielli E Orlando F Effect of the combination of clarithromycin and amikacin on Pseudomonas aeruginosa biofilm in an animal model of ureteral stent infection J Antimicrob Chemother 2011 66 06 1318 1323 10.1093/jac/dkr107 21406436

[14] Stoffel C Eltz B Salles M J Role of coatings and materials of external fixation pins on the rates of pin tract infection: A systematic review and meta-analysis World J Orthop 2021 12 11 920 930 10.5312/wjo.v12.i11.920 34888152 PMC8613683

[15] Saithna A The influence of hydroxyapatite coating of external fixator pins on pin loosening and pin track infection: a systematic review Injury 2010 41 02 128 132 10.1016/j.injury.2009.01.001 19486974

[16] Davies R Holt N Nayagam S The care of pin sites with external fixation J Bone Joint Surg Br 2005 87 05 716 719 10.1302/0301-620X.87B5.15623 15855378

[17] Britten S Ghoz A Duffield B Giannoudis P V Ilizarov fixator pin site care: the role of crusts in the prevention of infection Injury 2013 44 10 1275 1278 10.1016/j.injury.2013.07.001 23910230

[18] Stoffel C de Lima E Salles M J Hydroxyapatite-coated compared with stainless steel external fixation pins did not show impact in the rate of pin track infection: a multicenter prospective study Int Orthop 2023 47 05 1163 1169 10.1007/s00264-023-05717-w 36773051 PMC9918829

[19] Stoffel C L Complicações infecciosas no trajeto dos pinos de fixadores externos com e sem revestimento por hidroxiapatita – estudo prospectivo comparativo [tese] São Paulo Faculdade de Ciências Médicas da Santa Casa de São Paulo 2022 https://fcmsantacasasp.edu.br/wp-content/uploads/2022/07/2022-Cristhopher-Lucca-Stoffel_Final.pdf

[20] Clinical and Laboratory Standards Institute (CLSI) Standardization of Antimicrobial Disk Diffusion Susceptibility Testing: Approved Standard - Eighth Edition Wayne, PA CLSIdocument M02–A08, 2010, Vol. 23, No. 1

[21] Pieske O Kaltenhauser F Pichlmaier L Schramm N Trentzsch H Löffler T Clinical benefit of hydroxyapatite-coated pins compared with stainless steel pins in external fixation at the wrist: a randomised prospective study Injury 2010 41 10 1031 1036 10.1016/j.injury.2010.03.030 20444448

[22] McEvoy J P Martin P Khaleel A Dissanayeke S Titanium Kirschner wires resist biofilms better than stainless steel and hydroxyapatite-coated wires: an *in vitro* study Strateg Trauma Limb Reconstr 2019 14 02 57 64 10.5005/jp-journals-10080-1426 PMC7376582 32742415

[23] Oga M Arizono T Sugioka Y Bacterial adherence to bioinert and bioactive materials studied in vitro Acta Orthop Scand 1993 64 03 273 276 10.3109/17453679308993623 8391746

[24] Ravn C Ferreira I S Maiolo E Overgaard S Trampuz A Microcalorimetric detection of staphylococcal biofilm growth on various prosthetic biomaterials after exposure to daptomycin J Orthop Res 2018 36 10 2809 2816 10.1002/jor.24040 29744925

[25] Arciola C R Montanaro L Moroni A Giordano M Pizzoferrato A Donati M E Hydroxyapatite-coated orthopaedic screws as infection resistant materials: in vitro study Biomaterials 1999 20 04 323 327 10.1016/s0142-9612(98)00168-9 10048404

[26] W-Dahl A Toksvig-Larsen S Infection prophylaxis: a prospective study in 106 patients operated on by tibial osteotomy using the hemicallotasis technique Arch Orthop Trauma Surg 2006 126 07 441 447 10.1007/s00402-006-0165-y 16810553

